# MiR-200c-3p inhibits LPS-induced M1 polarization of BV2 cells by targeting RIP2

**DOI:** 10.1007/s13258-021-01210-z

**Published:** 2022-01-10

**Authors:** Lei Zhao, Xiaosong Liu, Jiankai Yang, Xiaoliang Wang, Xiaomeng Liu, Jianliang Wu, Chen Li, Donggang Xu, Yuhua Hu

**Affiliations:** 1 Department of Neurosurgery, The Second Hospital of Hebei Medical University, 215 Heping West Road, 050000 Shijiazhuang, Hebei China; 2 Institute of Military Cognition and Brain Science Research, Academy of Military Medical Sciences, Beijing, China

**Keywords:** LPS treatment, miR-200c-3p, RIP2, Microglia activation

## Abstract

**Background:**

Microglia are important immune cells, which can be induced by lipopolysaccharide (LPS) into M1 phenotype that express pro-inflammatory cytokines. Some studies have shown that microRNAs play critical roles in microglial activation.

**Objective:**

This study was designed to investigate the role of miR-200c-3p in regulating inflammatory responses of LPS-treated BV2 cells.

**Methods:**

The expression of miR-200c-3p in BV2 cells was detected by real-time PCR. Receptor-interacting protein 2 (RIP2) was predicted as a target gene of miR-200c-3p. Their relationship was verified by dual-luciferase reporter assay. The function of miR-200c-3p and RIP2 in microglial polarization and NF-κB signaling was further evaluated.

**Results:**

LPS treatment reduced miR-200c-3p expression in a dose-dependent and time-dependent manner in BV2 cells. LPS treatment increased the expression of M1 phenotype markers inducible nitric oxide synthase (iNOS) and major histocompatibility complex class (MHC)-II, promoted the release of pro-inflammatory cytokines interleukin (IL)-1β, IL-6 and tumor necrosis factor (TNF)-α, and enhanced the nuclear translocation and phosphorylation of nuclear factor-kappaB (NF-κB) p65. Reversely, miR-200c-3p mimics down-regulated the levels of these inflammatory factors. Furthermore, RIP2 was identified to be a direct target of miR-200c-3p. RIP2 knockdown had a similar effect to miR-200c-3p mimics. Overexpression of RIP2 eliminated the inhibitory effect of miR-200c-3p on LPS-induced M1 polarization and NF-κB activation in BV2 cells.

**Conclusions:**

MiR-200c-3p mimics suppressed LPS-induced microglial M1 polarization and NF-κB activation by targeting RIP2. MiR-200c-3p/RIP2 might be a potential therapeutic target for the treatment of neuroinflammation-associated diseases.

**Supplementary Information:**

The online version contains supplementary material available at 10.1007/s13258-021-01210-z.

## Introduction

Microglia are a unique group of cells in central nervous system that originate in myeloid system. In the brain, they are generally recognized as the resident immune cells (Vilhardt 2005). Microglia are usually at a resting state, but may be activated at functional polarization states (classically activated M1 type and alternatively activated M2 type) after infection or injury (Li et al. 2015). The M1-polarized microglia produce a variety of proinflammatory factors, such as reactive oxygen species, prostaglandin E2, nitric oxide and proinflammatory cytokines tumor necrosis factor (TNF)-α, interleukin (IL)-6 and IL-1β, and other potentially neurotoxic compounds (Zheng et al. 2018a). Studies have shown that the over-activation of microglia may accelerate the process of some central nervous system diseases, and the release of cytotoxic factors may lead to further damage of neurons (Eren et al. 2018). Therefore, controlling the activation of microglia may help to improve neuron survival (Yao et al. 2018).

MicroRNAs (miRNAs) are a class of single stranded non-coding RNAs that regulate gene expression by binding to their 3’untranslated region (UTR) at post-transcriptional level (Lv et al. 2017; Yin et al. 2017). MiR-200c-3p is a member of the miR-200 family (Liu et al. 2017). Some functions of the miR-200 family have been reported. For example, miR-200b is reported to target zinc finger E-box-binding homeobox 1 (ZEB1) to promote cell apoptosis (Filios et al. 2014). In leiomyoma smooth muscle cells, gain-of function of miR-200c-3p repressed nuclear factor κB (NF-κB) pathway by targeting inhibitor of nuclear factor κB kinase subunit B (IKBKB), thereby reducing the ability of NF-κB p65 binding to IL-8 promoter (Chuang and Khorram 2014). In addition, miR-200c-3p has been reported to exert regulatory effects on biological processes in different cells (Chen et al. 2021; Hu et al. 2021; Jiang et al. 2020; Liu et al. 2018), but the role of miR-200c-3p in microglia has not been reported.

Receptor-interacting protein 2 (RIP2, also known as RIPK2) is a serine/threonine kinase. RIP2 is consisted of a N-terminal kinase domain, an intermediate domain, a C-terminal caspase activation and recruitment domain (Jaafar et al. 2018). It functions as one of the mediators of lipopolysaccharide (LPS) dependent activation in intracellular signaling pathways (Usluoglu et al. 2007). RIP2 knockdown inhibited the expression of p40, a subunit of LPS-dependent proinflammatory cytokine IL-12 (Usluoglu et al. 2007). In addition, LPS-treated RIP2-deficient mice showed impaired activation of NF-κB and reduced cytokines production (Lu et al. 2005). RIP2 is a downstream adapter of the intracellular recognition receptor nucleotide-binding oligomerisation domain 1 (NOD1). NOD1/RIP2 signaling can induce the production of inflammatory cytokines (Wang et al. 2020b). Obviously, RIP2 plays an important role in regulating inflammation and innate immunity.

In this study, we examined the effect of miR-200c-3p on microglia activation in LPS-stimulated BV2 cells *in vitro*. Based on website predictions, we speculated that RIP2 may be targeted by miR-200c-3p. We explored the role of miR-200c-3p and RIP2 in microglia activation. It might provide a potential new strategy for the treatment of neuroinflammatory diseases.

## Materials and methods

### Microglia culture and LPS treatment

Mice BV2 cells (iCell Bioscience Inc, China) were cultured in DMEM medium (hyclone, USA) containing 10% fetal bovine serum (Biological Industries, Israel) and 1% GlutaMAX (Gibco, USA) in an incubator (Shanghai Lishen Scientific Equipement Co., Ltd, China) at 37 ℃ and 5% CO_2_. BV2 cells were treated with 0, 10, 100 and 1000 ng/mL LPS (Solarbio, China) for 24 h or 10 ng/mL LPS for 0, 4, 12 and 24 h.

### RNA isolation and real-time PCR

Total RNA with miRNAs and mRNAs was extracted from BV2 cells using RNApure total RNA fast isolation kit (BioTeke, China) according to the manufacturer’s instructions. The mRNA in the samples was reversely transcribed by BeyoRT™ II M-MLV reverse transcriptase (Beyotime, China) to produce cDNA. The miRNA was reversely transcribed by miRNA first strand cDNA synthesis (Tailing Reaction) (Sangon, China) to produce cDNA. Real-time PCR was performed using SYBR Green (BioTeke, China) on an Exicycler^TM^ 96 real-time thermal block (Bioneer, Korea) according to the manufacturer’s procedure. The following specific primers were used for real-time PCR.

RIP2, forward primer: 5′-CTCCTCGTGTTCCTTGGC-3′, reverse primer: 5′- TGGCTCACAATGGCTTCC-3′;

iNOS, forward primer: 5′-CACCACCCTCCTCGTTC-3′, reverse primer: 5′- CAATCCACAACTCGCTCC-3′;

MHC-II, forward primer: 5′-CACCCTCATCTGCTTTGT-3′, reverse primer: 5′- TCAGGTTCCCAGTGTTTC-3′;

The 2^−ΔΔCT^ formula was used for relative quantitative calculation (Magro et al. 2007). β-actin was used as the internal reference gene for standardization. All experiments are conducted in triplicate.

### Dual-luciferase reporter assay

According to the website (http://www.targetscan.org/vert_72/) prediction, a potential target site of miR-200c-3p (nucleotides 138-145 bp) was found on the 3’UTR (297 bp) of RIP2. The 3’UTR containing wt (GCAGTATT) or mutated RIP2 target site (CGTCATAA) was inserted into the luciferase reporter plasmid pmirGLO (Promega, China). 293T cells (Shanghai Zhong Qiao Xin Zhou Biotechnology Co., Ltd, China) were seeded into 24-well plates and co-transfected with 250 ng luciferase reporter plasmid and 10 pmol miR-200c-3p mimics using lipofectamine 2000 (invitrogen, USA). After 48 h, the luciferase activity was measured using the dual luciferase reporter gene assay kit (KeyGen, China) in accordance with the manufacturer’s instructions. The luciferase activity of firefly in each transfection well was normalized to renilla luciferase activity. Each sample was set to three replicates.

### Western blot analysis

The total protein was extracted using RIPA lysis buffer (Beyotime, China). Nuclear protein was extracted using nuclear and cytoplasmic protein extraction kit (Beyotime, China). The protein concentration was quantified using BCA protein assay kit (Beyotime, China) according to the manufacturer’s instructions. Protein was separated and transferred to PVDF membranes (Thermo Scientific, USA). These membranes were blocked with 5% (M/V) BSA (Biosharp, China) for 1 h and incubated with indicated primary antibodies at 4 ℃ overnight. The membranes were then incubated at 37 ℃ for 40 min with secondary antibodies including HRP-conjugated affinipure goat anti-mouse IgG (H+L) (1:10,000, SA00001-1, proteintech, China) or HRP-conjugated affinipure goat anti-rabbit IgG (H+L) (1:10,000, SA00001-2, proteintech, China). The protein blots were observed using ECL reagents (7 Sea biotech, China) and captured using a gel image processing system (Gel-Pro-Analyzer software) (Beijing Liuyi Biotechnology Co., Ltd, China). The primary antibodies used are as follows:

RIP2 antibody (1:500, A2498, ABclonal, China), p-p65 (Ser 536) antibody (1:1000, AP0124, ABclonal, China), p65 antibody (1:1000, A19653, ABclonal, China), Histone-H3 antibody (1:500, 17168-1-AP, proteintech, China) and beta actin antibody (1:2000, 60008-1-Ig, proteintech, China).

### Immunofluorescence

The sections were fixed in 4% paraformaldehyde (Sinopharm Chemical Reagent Co., Lid, China) for 15 min and penetrated with 0.1% TritonX-100 (Beyotime, China). Sections were incubated with goat serum (Solarbio, China) at room temperature for 15 min. Then sections were incubated with primary antibody p65 (1:200, A11201, ABclonal, China) at 4 ℃ overnight. After washing, the sections were incubated with Cy3-labeled goat anti-rabbit IgG (H+L) (1:200, A0516, Beyotime, China) at room temperature for 60 min. After removing the secondary antibody, 4′,6-diamidino-2-phenylindole (DAPI) (Beyotime, China) was added to the sections for nucleus staining. Finally, mounting medium (Solarbio, China) was dropped on the slide. The staining results were observed under the fluorescence microscope (Olympus, Japan).

### Enzyme-linked immune-sorbent assay

The contents of TNF-α, IL-1β, and IL-6 in cell culture supernatants were measured using ELISA kits (Lianke Biotech, Co., Ltd, China) according to the manufacturer’s instructions. The absorbance at 450 nm and 570 nm was measured with a microplate analyzer (Biotek, USA), and the calibrated OD value was 450 nm minus 570 nm.

### Flow cytometry

The cells were collected, resuspended with PBS and incubated with anti-mouse CD86 (10^6^ cells/5 µL, Lianke Biotech, Co., Ltd, China) at 4 ℃ for 30 min in darkness. The cells were then washed with 1 mL PBS. A NovoCyte flow cytometer (Aceabio, USA) was used for detection.

### siRNA and plasmid preparation

RIP2 siRNA-1 sequences 5′-GCUAAAGAAAGCAAAGAUATT-3′, 5′- UAUCUUUGCUUUCUUUAGCTT-3′, RIP2 siRNA-2 sequences 5′- CAGAGUUCCUCAAGUACUATT-3′, 5′-UAGUACUUGAGGAACUCUGTT-3′, NC siRNA sequences 5′- UUCUCCGAACGUGUCACGUTT-3′, 5′ ACGUGACACGUUCGGAGAATT-3′. The full-length mouse RIP2 CDS clone was used for overexpression of RIP2, vector was as the control.

### Cell transfection

BV2 cells were cultured in an incubator at 37 ℃ and 5% CO_2_. MiR-200c-3p mimics, RIP2 OE (RIP2 CDS clone) or RIP2 siRNA was used to transfect with cells. Transfection was performed when the cell density was 60-70%. The transfection reagents were as follows:

Solution 1: 125 µL Opti-MEM (invitrogen, USA) + 9 µL lipofectamine 2000 (invitrogen, USA), mixed.

Solution 2: 125 µL Opti-MEM +100 pmoL miR-200c-3p mimics, 100 pmoL RIP2 siRNA or 2.5 µg RIP2-OE, mixed.

The solution is slowly dripped into the cells and gently shaken. The cells were cultured in an incubator at 37 ℃ and 5% CO_2_.

### Statistical analysis

Data were analyzed by using GraphPad Prism 8.0 software. The experimental data were represented as mean ± standard deviation. Multiple comparisons were analyzed using one-way analysis of variance followed by Tukey’s test. The value of *p* < 0.05 was considered statistically significant.

## Results

### MiR-200c-3p levels in LPS-treated BV2 cells

**Fig. 1:**
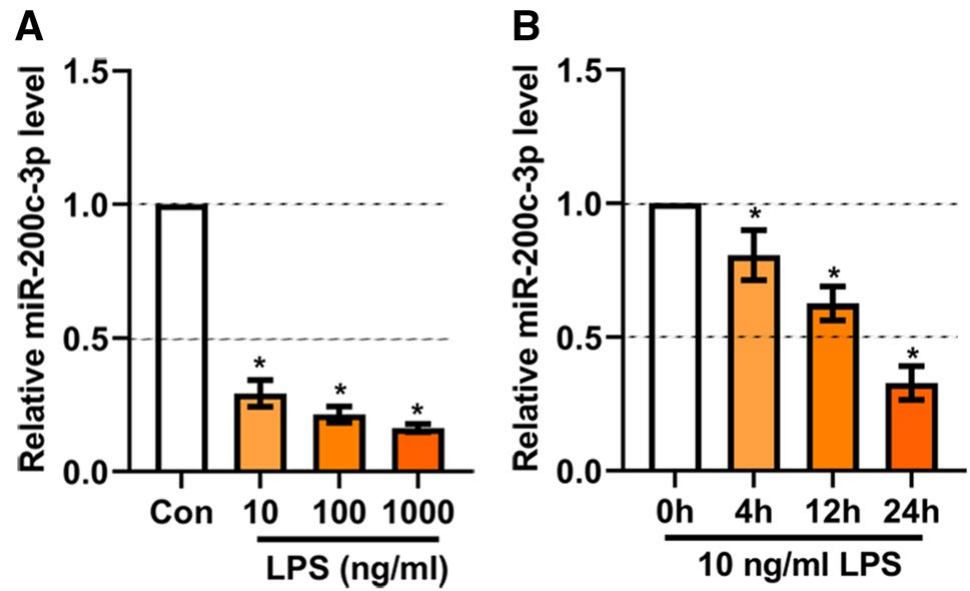
MiR-200c-3p levels in LPS-treated BV2 cells. **A** MiR-200c-3p levels in BV2 cells after LPS treatment at different concentrations for 24 h. *p  < 0.05 vs. Con group. Con: Control. **B** MiR-200c-3p levels in BV2 cells after 10 ng/ml LPS treatment for different time period. *p < 0.05 vs. 0 h. Data were represented as mean ± SD (n = 3) and analyzed by one-way analysis of variance (ANOVA) followed by Tukey’s multiple comparison test

After treatment of indicated concentration of LPS, the expression level of miR-200c-3p was detected. We found that LPS induced a significant downregulated expression of miR-200c-3p in a dosage-dependent manner (*p* < 0.05) (Fig. 1A). In addition, we also detected the expression of miR-200c-3p at different time points after the treatment of 10 ng/mL LPS. As shown in Fig. 1B, LPS induced a time-dependent downregulation of miR-200c-3p from 0 to 24 h.

### MiR-200c-3p mimics inhibited M1 polarization in LPS-treated BV2 cells

**Fig. 2:**
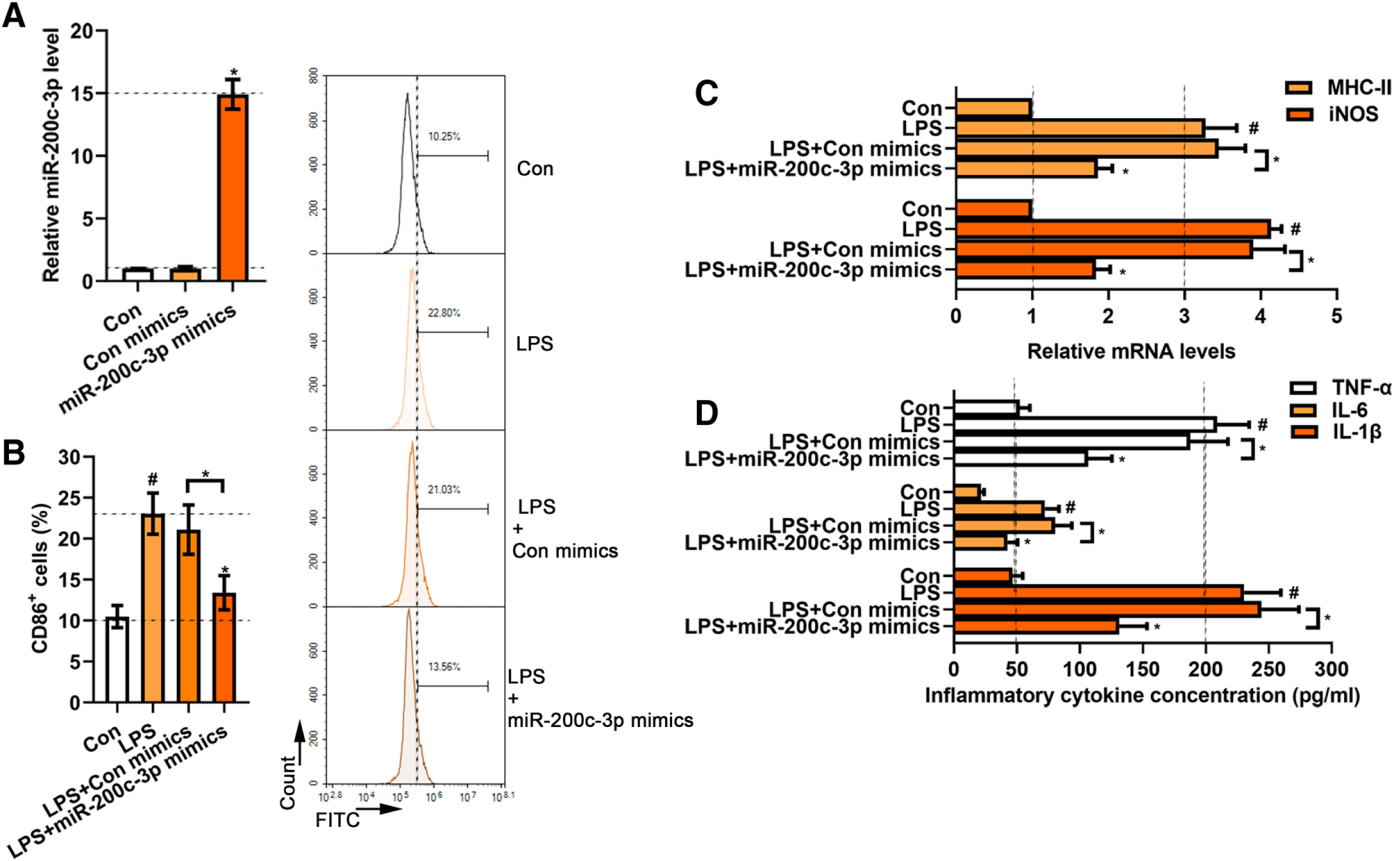
Effects of miR-200c-3p on microglia activation in LPS-treated BV2 cells. **A** MiR-200c-3p level in BV2 cells after miR-200c-3p mimics transfection. **B** Flow cytometry was used to analyze CD86 positive cell percentage in LPS-treated BV2 cells with miR-200c-3p mimics transfection. **C** Real-time PCR was used to assess MHC-II and iNOS mRNA expression in LPS-treated BV2 cells with miR-200c-3p mimics transfection. **D** The generation of TNF-α, IL-6 and IL-1β in LPS-induced BV2 cells with miR-200c-3p mimics transfection. *Con* Control; *Con mimics* Control mimics. Data were represented as mean ± SD (n = 3) and analyzed by one-way analysis of variance (ANOVA) followed by Tukey’s multiple comparison test. ^#^*p* < 0.05 vs. Con group; *p < 0.05 vs. Con mimics; *p < 0.05 vs. LPS or the indicated group

To investigate whether miR-200c-3p functions in LPS-treated BV2 cells, we transfected BV2 cells with miR-200c-3p mimics. Real-time PCR showed that miR-200c-3p mimics significantly increase the expression of miR-200c-3p (*p* < 0.05) (Fig. 2 A). Flow cytometry analysis showed that LPS (10 ng/mL) treatment for 24 h significantly increased the percentage of CD86^+^ BV2 cells upon the control cells (*p* <0.05) (Fig. 2B). In contrast, miR-200c-3p significantly reduced the percentage of CD86^+^ BV2 cells (*p* < 0.05) (Fig. 2B). Next, we examined the mRNA expression levels of iNOS and MHC-II, which are markers of microglial M1 polarization (Aryanpour et al. 2017). Compared with the control cells, mRNA expression levels of iNOS and MHC-II in LPS-treated BV2 cells were significantly increased (*p* < 0.05), while miR-200c-3p mimics significantly decreased the expression levels of these factors (*p* < 0.05, Fig. 2C). We further detected the release of inflammatory factors by employing ELISA. LPS treatment significantly increased the levels of inflammatory cytokines IL-1β, IL-6 and TNF-α in cell supernatant (*p* < 0.05), but these increases were reversed by miR-200c-3p mimics (*p* < 0.05) (Fig. 2D). These data suggested that miR-200c-3p inhibited the M1 polarization of LPS-treated BV2 cells.

### MiR-200c-3p inhibited NF-κB activation in LPS-treated BV2 cells

**Fig. 3:**
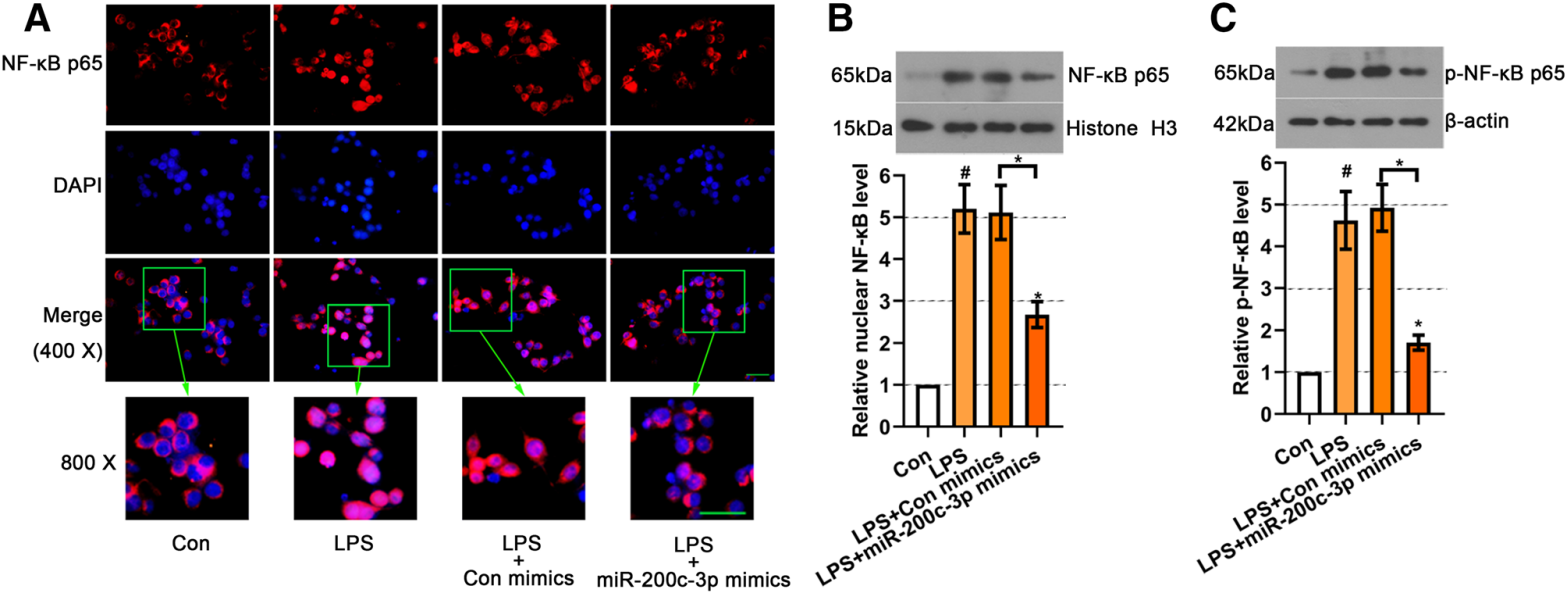
Effects of miR-200c-3p on NF-κB activation in LPS-treated BV2 cells. **A** The expression of NF-κB p65 was examined by immunofluorescence staining at 400 × and 800 × magnification in LPS-treated BV2 cells with miR-200c-3p mimics transfection. Representative western blot for **B** nuclear NF-κB p65 and **C** p-NF-κB p65 in LPS-treated BV2 cells with miR-200c-3p mimics transfection. Data were represented as mean ± SD (n = 3) and analyzed by one-way analysis of variance (ANOVA) followed by Tukey’s multiple comparison test. ^#^*p* < 0.05 vs. Con group; *p < 0.05 vs. LPS or the indicated group

NF-κB is an important signal transduction factor in central nervous system diseases. It promotes the expression of many chemokines and pro-inflammatory cytokines in microglia (Doyle and O’Neill 2006). We further investigate the role of miR-200c-3p in LPS-induced NF-κB activation in BV2 cells. As shown in Fig. 3A, LPS treatment induced nuclear translocation of NF-κB p65, which was obviously suppressed by miR-200c-3p mimics. The results of western blot analysis also reflected that the increased nuclear expression and phosphorylation level of NF-κB p65 induced by LPS were remarkably decreased in miR-200c-3p mimics transfected BV2 cells (*p* < 0.05, Fig. 3B, C).

### RIP2 was the target gene of miR-200c-3p

**Fig. 4:**
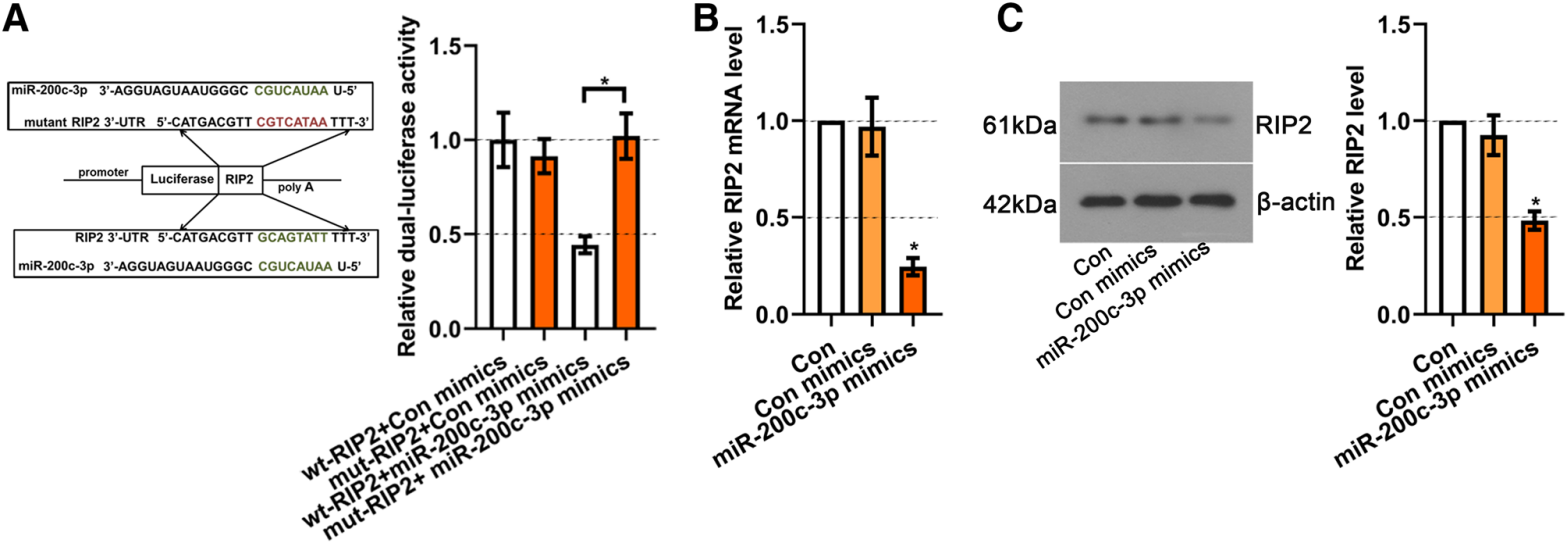
RIP2 was the target gene of miR-200c-3p. **A** Dual-Luciferase reporter assay was performed to detect the binding between RIP2 3’UTR and miR-200c-3p in HEK 293T cells. **B** Real-time PCR was used to assess RIP2 mRNA expression in BV2 cells with miR-200c-3p mimics transfection. **C** Western blot for RIP2 in BV2 cells with miR-200c-3p mimics transfection. Data were represented as mean ± SD (n = 3) and analyzed by one-way analysis of variance (ANOVA) followed by Tukey’s multiple comparison test. *p < 0.05 vs. Con mimics group or the indicated group

Targetscan website provides a putative binding site for miR-200c-3p in the 3’UTR of RIP2 (Fig. 4A). Dual-luciferase analysis showed that the miR-200c-3p mimics significantly restrained the luciferase activity of RIP2 3’UTR reporter gene (wt-RIP2), but had no effect on the seed region mutants (mut-RIP2) (*p* <0.05) (Fig. 4A). Then we detected the mRNA expression of RIP2 in miR-200c-3p mimics-transfected cells and found it was dramatically decreased compared with the cells transfected with control mimics (*p* < 0.05) (Fig. 4B). Western blot results also showed the significant decreased RIP2 protein expression in the miR-200c-3p mimics-transfected cells (*p* <0.05) (Fig. 4C). These results suggested that miR-200c-3p directly binds to 3’UTR of RIP2 and regulated the expression of RIP2 in BV2 cells.

### RIP2 knockdown suppressed microglia activation in LPS-treated BV2 cells

**Fig. 5:**
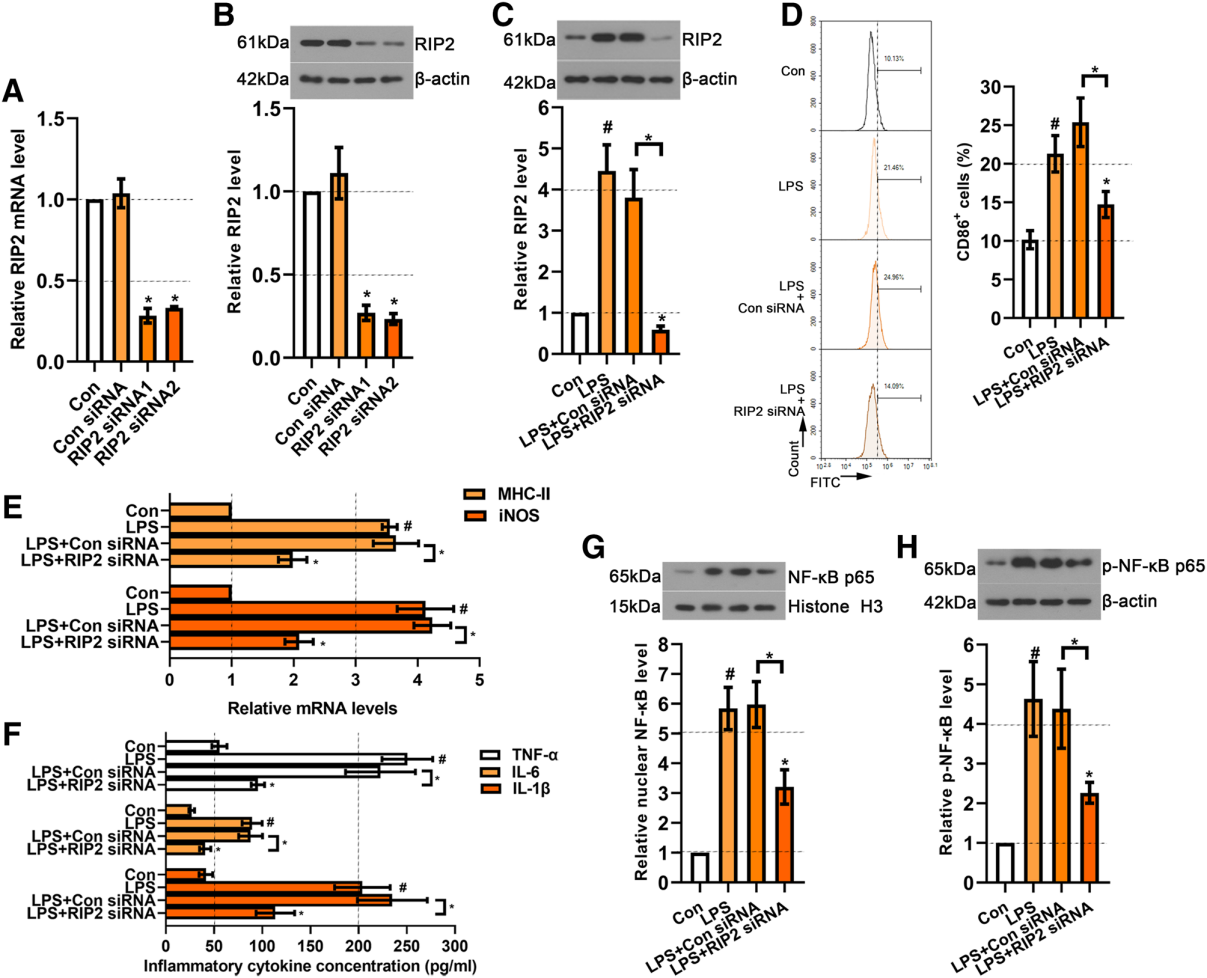
Effects of RIP2 knockdown on microglial activation in LPS-treated BV2 cells. **A** Real-time PCR was used to assess RIP2 mRNA expression in BV2 cells with RIP2 siRNA transfection. **B** Western blot for RIP2 in BV2 cells with RIP2 siRNA transfection. **C** Western blot for RIP2 in LPS-treated BV2 cells with RIP2 siRNA transfection. **D** Flow cytometry was used to analyze CD86 positive cell percentage in LPS-treated BV2 cells with RIP2 siRNA transfection. **E** Real-time PCR was used to assess MHC-II and iNOS mRNA expression in LPS-treated BV2 cells with RIP2 siRNA transfection. **F** The generation of TNF-α, IL-6 and IL-1β in LPS-induced BV2 cells with RIP2 siRNA transfection. Representative western blot for **G** nuclear NF-κB p65 and **H** p-NF-κB p65 in LPS-induced BV2 cells with RIP2 siRNA transfection. Data were represented as mean ± SD (n = 3) and analyzed by one-way analysis of variance (ANOVA) followed by Tukey’s multiple comparison test. ^#^*p* < 0.05 vs. Con group; *p < 0.05 vs. LPS or the indicated group

To determine the role of RIP2 knockdown in microglia activation, we transfected siRNA into BV2 cells. Two siRNAs targeted RIP2 were obtained and named as RIP2 siRNA1 and RIP2 siRNA2. The knockdown efficiency was evaluated by real-time PCR and western blot. The results showed that the siRNAs successfully mediated RIP2 knockdown in BV2 cells (*p* <  0.05) (Fig. 5A, B). Subsequently, a siRNA with high transfection efficiency was selected to use in the following experiments. Intriguingly, we observed that LPS treatment elevated the RIP2 expression in BV2 cells, whereas RIP2 siRNA reduced its expression (Fig. 5C). In addition, as illustrated in Fig. 5D, flow cytometry showed that RIP2 siRNA significantly decreased the percentage of CD86^+^ cells in LPS-treated BV2 cells (*p* < 0.05). The same effects were also found in the mRNA expression levels of iNOS and MHC-II, release of IL-1β, TNF-α and IL-6. All these enhanced M1 polarization markers and pro-inflammatory cytokines induced by LPS were restored by RIP2 siRNA (Fig. 5E, F). Furthermore, RIP2 siRNA also inhibited LPS-induced p65 nuclear translocation and phosphorylation level (Fig. 5G, H). These results suggested that RIP2 participated in the M1 polarization of LPS-induced BV2 cells.

### MiR-200c-3p suppressed microglia activation in LPS-treated BV2 cells via RIP2

**Fig. 6:**
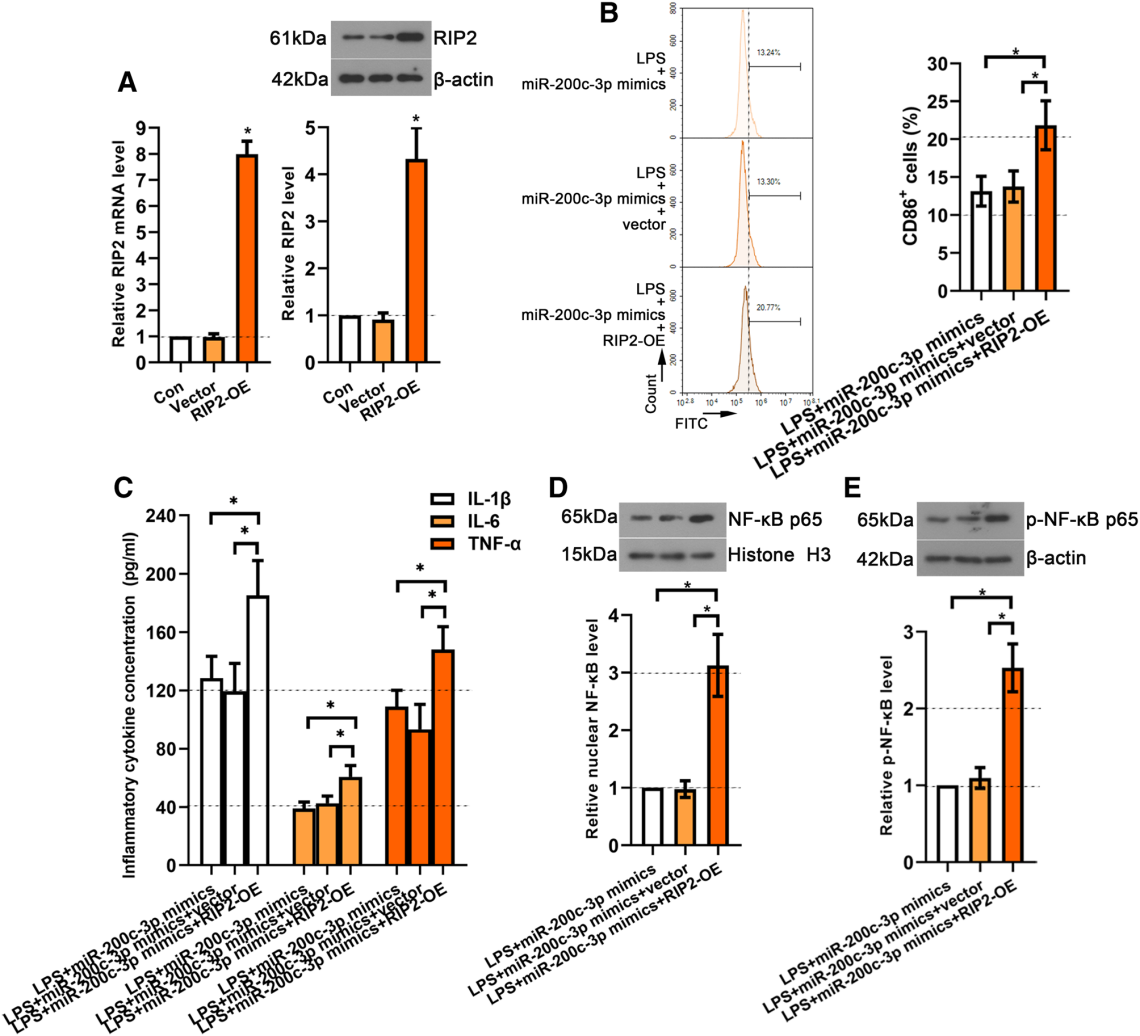
RIP2 overexpression eliminated the inhibiting effects of miR-200c-3p on microglial activation in LPS-treated BV2 cells. **A** Real-time PCR and western blot were used to assess RIP2 mRNA and protein expression in BV2 cells with RIP2 overexpressed plasmid transfection. **B** Flow cytometry was used to analyze CD86 positive cell percentage in LPS-treated BV2 cells with miR-200c-3p mimics and RIP2 overexpressed plasmid co-transfection. **C** The generation of TNF-α, IL-6 and IL-1β in LPS-treated BV2 cells with miR-200c-3p mimics and RIP2 overexpressed plasmid co-transfection. Representative western blot for **D** nuclear NF-κB p65 and **E** p-NF-κB p65 in LPS-treated BV2 cells with miR-200c-3p mimics and RIP2 overexpressed plasmid co-transfection. Data were represented as mean ± SD (n = 3) and analyzed by one-way analysis of variance (ANOVA) followed by Tukey’s multiple comparison test. *p < 0.05 vs. the vector group; *p < 0.05 vs. the indicated group

To evaluate whether miR-200c-3p/RIP2 affects microglia activation in LPS-treated BV2 cells, miR-200c-3p mimics were co-transfected with RIP2 overexpression (RIP2-OE) plasmid into LPS-treated BV2 cells. Real-time PCR and western blot showed that the mRNA and protein expression levels of RIP2 in RIP2-OE group were significantly increased (*p* < 0.05) (Fig. 6A). Flow cytometry result illustrated that miR-200c-3p mimics + RIP2-OE significantly increased the percentage of CD86^+^ cells in LPS-treated BV2 cells compared with miR-200c-3p mimics + vector group (*p* <0.05) (Fig. 6B). Furthermore, miR-200c-3p mimics + RIP2-OE significantly increased the contents of pro-inflammatory cytokines TNF-α, IL-1β, and IL-6 (*p* < 0.05) (Fig. 6C). Furthermore, miR-200c-3p mimics + RIP2-OE also enhanced LPS-induced p65 nuclear translocation and phosphorylation level (*p* <0.05) (Fig. 6D, E). The results showed that miR-200c-3p suppressed microglia activation in LPS-treated BV2 cells by mediating RIP2.

## Discussion

MiRNAs have been reported to modulate neuronal and immune processes (Chen et al. 2007, 2019; Lind et al. 2013). MiR-200c-3p (previously named miR-200c) inhibits the expression of pro-inflammatory factors including IL-6, IL-8 and CCL-5, and improves osteogenic differentiation (Hong et al. 2016). Overexpression of miR-200c-3p in MDA-MB-231 cell line can up-regulate the expression of CD206 in RAW264.7 cells, and it may promote the M2 polarization of macrophages (Meng et al. 2020). Also, lncRNA X-inactive specific transcript promoted apoptosis and inflammatory response stimulated by cigarette smoke extract through miR-200c-3p (Chen et al. 2021). However, the role of miR-200c-3p in microglia activation has not been reported. In our research, we demonstrated that miR-200c-3p suppressed M1 polarization of LPS-treated BV2 cells, accompanying with NF-κB inhibition and reduction of pro-inflammatory cytokines secretion.

Microglia, as important immune cells in central nervous system, can switch phenotype (M1/M2) in response to the change of microenvironment (Xiong et al. 2016). In response to injury or brain damage, microglia are activated and undergo morphological and functional changes (Nimmerjahn et al. 2005). Activated microglia affect survival of neurons through the release of pro-inflammatory factors such as TNF-α, IL-1β and IL-6 (Kong et al. 2014). These pro-inflammatory cytokines play vital roles in mediating immune responses (Freeman and Ting 2016). In the present study, we found that LPS treatment enhanced the expression of iNOS and MHC-II and released pro-inflammatory factors TNF-α, IL-1β and IL-6 in LPS-treated BV2 cells. MiR-200c-3p suppressed the elevated levels of these factors. Moreover, NF-κB is a critical signal transduction factor in central nervous system diseases, and may mediate the production of many chemokines and cytokines in microglia (Doyle and O’Neill 2006). In our study, we consistently demonstrated that miR-200c-3p mimics inhibited LPS-induced activation on NF-κB signaling pathway. All these findings suggested that miR-200c-3p had an inhibitory effect on LPS-induced microglial activation.

To explore the potential mechanism of miR-200c-3p in affecting LPS-treated BV2 cells activation, we searched the target genes that miR-200c-3p may affect. RIP2 was predicted to be a putative target gene of miR-200c-3p. RIP2 plays an important role in innate and adaptive immunity (Jaafar et al. 2018). In primary microglia, RIP2 inhibition partially alleviated intracerebral haemorrhage-induced brain injury by reducing microglia activation (Wang et al. 2020a). In our study, miR-200c-3p was validated to complementarily bind to RIP2 3’UTR and negatively regulate RIP2 mRNA and protein expression in BV2 cells. These results suggested that miR-200c-3p mediated RIP2 post-transcriptional regulation in BV2 cells.

RIP2 was reported to mediate proinflammatory response. A previous study reported that pattern recognition receptor nucleotide-binding oligomerization domain-containing 2 (NOD2) activation enhances the expression of pro-inflammatory cytokines IL-6, TNF-α, IL-1β and IFN-γ in a RIP2-dependent manner (Zhou et al. 2021). RIP2 was significantly increased after 24 h stimulation by the pro-inflammatory cytokines IL-1β or TNF-α (Wang et al. 2020a). Furthermore, suppression of RIP2 impaired proinflammatory cytokines production in mouse brain tissue after pneumococcal meningitis infection (Zheng et al. 2018b). Consistently in our study, we also explored the role of RIP2 knockdown in LPS-treated BV2 cells. We found that RIP2 knockdown inhibited M1 polarization of LPS-treated BV2 cells by inhibiting microglial M1 polarization and reducing pro-inflammatory cytokines secretion. In addition, RIP2 knockdown also suppressed NF-κB signaling pathway in LPS-treated BV2 cells.

We further investigated the correlation of miR-200c-3p and RIP2 in activation and inflammation response of LPS-treated BV2 cells. We found that miR-200c-3p mimics transfection suppressed RIP2 expression. To investigate whether miR-200c-3p affects microglia by regulating RIP2, we performed a rescue experiment. As we expected, overexpression of RIP2 partially abolished the inhibitory effect of miR-200c-3p on microglial activation, as reflected in aspects of inflammation, M1 polarization and NF-κB signaling.

In conclusion, miR-200c-3p participates in microglial M1 polarization and inflammatory responses. MiR-200c-3p mimics inhibited LPS-induced microglial M1 polarization by directly targeting RIP2. Our findings provide a novel understanding of miR-200c-3p/RIP2 in microglial M1 polarization and it may be a possible therapeutic target for neuroinflammation-associated diseases.

## Electronic Supplementary Material

Below is the link to the electronic supplementary material.

Supplementary Material 1 (DOCX 16 KB)

## Data Availability

All data generated or analyzed during this study are included in this article.

## References

[CR1] Aryanpour R, Pasbakhsh P, Zibara K, Namjoo Z, Beigi Boroujeni F, Shahbeigi S, Kashani IR, Beyer C, Zendehdel A (2017). Progesterone therapy induces an M1 to M2 switch in microglia phenotype and suppresses NLRP3 inflammasome in a cuprizone-induced demyelination mouse model. Int Immunopharmacol.

[CR2] Chen L, Dong R, Lu Y, Zhou Y, Li K, Zhang Z, Peng M (2019). MicroRNA-146a protects against cognitive decline induced by surgical trauma by suppressing hippocampal neuroinflammation in mice. Brain Behav Immun.

[CR3] Chen P, Jiang P, Chen J, Yang Y, Guo X (2021). XIST promotes apoptosis and the inflammatory response in CSE-stimulated cells via the miR-200c-3p/EGR3 axis. BMC Pulm Med.

[CR4] Chen X-M, Splinter PL, O’Hara. SP, LaRusso. NF (2007). A cellular micro-RNA, let-7i, regulates Toll-like receptor 4 expression and contributes to cholangiocyte immune responses against Cryptosporidium parvum infection. J Biol Chem.

[CR5] Chuang TD, Khorram O (2014). miR-200c regulates IL8 expression by targeting IKBKB: a potential mediator of inflammation in leiomyoma pathogenesis. PLoS One.

[CR6] Doyle SL, O’Neill LA (2006). Toll-like receptors: from the discovery of NFkappaB to new insights into transcriptional regulations in innate immunity. Biochem Pharmacol.

[CR7] Eren E, Tufekci KU, Isci KB, Tastan B, Genc K, Genc S (2018). Sulforaphane inhibits lipopolysaccharide-induced inflammation, cytotoxicity, oxidative stress, and miR-155 expression and switches to Mox phenotype through activating extracellular signal-regulated kinase 1/2-nuclear factor erythroid 2-related factor 2/antioxidant response element pathway in murine microglial cells. Front Immunol.

[CR8] Filios SR, Xu G, Chen J, Hong K, Jing G, Shalev A (2014). MicroRNA-200 is induced by thioredoxin-interacting protein and regulates Zeb1 protein signaling and beta cell apoptosis. J Biol Chem.

[CR9] Freeman LC, Ting JP (2016). The pathogenic role of the inflammasome in neurodegenerative diseases. J Neurochem.

[CR10] Hong L, Sharp T, Khorsand B, Fischer C, Eliason S, Salem A, Akkouch A, Brogden K, Amendt BA (2016). MicroRNA-200c represses IL-6, IL-8, and CCL-5 expression and enhances osteogenic differentiation. PLoS One.

[CR11] Hu X, Bai S, Li L, Tian P, Wang S, Zhang N, Shen B, Du J, Liu S (2021). MiR-200c-3p increased HDMEC proliferation through the notch signaling pathway. Exp Biol Med (Maywood).

[CR12] Jaafar R, Mnich K, Dolan S, Hillis J, Almanza A, Logue SE, Samali A, Gorman AM (2018). RIP2 enhances cell survival by activation of NF-ĸB in triple negative breast cancer cells. Biochem Biophys Res Commun.

[CR13] Jiang Y, Ji X, Liu K, Shi Y, Wang C, Li Y, Zhang T, He Y, Xiang M, Zhao R (2020). Exosomal miR-200c-3p negatively regulates the migraion and invasion of lipopolysaccharide (LPS)-stimulated colorectal cancer (CRC). BMC Mol Cell Biol.

[CR14] Kong H, Omran A, Ashhab MU, Gan N, Peng J, He F, Wu L, Deng X, Yin F (2014). Changes in microglial inflammation-related and brain-enriched MicroRNAs expressions in response to in vitro oxygen-glucose deprivation. Neurochem Res.

[CR200] Li L, Sun Q, Li Y, Yang Y, Yang Y, Chang T, Man M, Zheng L (2015). Overexpression of SIRT1 induced by resveratrol and inhibitor of miR-204 suppresses activation and proliferation of microglia. J Mol Neurosci.

[CR15] Lind EF, Elford AR, Ohashi PS (2013). Micro-RNA 155 is required for optimal CD8+ T cell responses to acute viral and intracellular bacterial challenges. J Immunol.

[CR16] Liu J, Wang L, Li X (2018). HMGB3 promotes the proliferation and metastasis of glioblastoma and is negatively regulated by miR-200b-3p and miR-200c-3p. Cell Biochem Funct.

[CR17] Liu Q, Du J, Yu X, Xu J, Huang F, Li X, Zhang C, Li X, Chang J, Shang D (2017). miRNA-200c-3p is crucial in acute respiratory distress syndrome. Cell Discov.

[CR18] Lu C, Wang A, Dorsch M, Tian J, Nagashima K, Coyle AJ, Jaffee B, Ocain TD, Xu Y (2005). Participation of Rip2 in lipopolysaccharide signaling is independent of its kinase activity. J Biol Chem.

[CR19] Lv YN, Ou-Yang AJ, Fu LS (2017). MicroRNA-27a negatively modulates the inflammatory response in lipopolysaccharide-stimulated microglia by targeting TLR4 and IRAK4. Cell Mol Neurobiol.

[CR20] Magro AM, Magro. AD CC, Miller. MR (2007). Down-regulation of vinculin upon MK886-induced apoptosis in LN18 glioblastoma cells. Neoplasma.

[CR21] Meng Z, Zhang R, Wang Y, Zhu G, Jin T, Li C, Zhang S (2020). miR-200c/PAI-2 promotes the progression of triple negative breast cancer via M1/M2 polarization induction of macrophage. Int Immunopharmacol.

[CR22] Nimmerjahn A, Kirchhoff F, Helmchen F (2005). Resting microglial cells are highly dynamic surveillants of brain parenchyma in vivo. Science.

[CR23] Usluoglu N, Moelling. PJ, Radziwill. K (2007). RIP2 mediates LPS-induced p38 and IkappaBalpha signaling including IL-12 p40 expression in human monocyte-derived dendritic cells. Eur J Immunol.

[CR201] Vilhardt F (2005). Microglia: phagocyte and glia cell. Int J Biochem Cell Biol.

[CR24] Wang M, Ye X, Hu J, Zhao Q, Lv B, Ma W, Wang W, Yin H, Hao Q, Zhou C (2020). NOD1/RIP2 signalling enhances the microglia-driven inflammatory response and undergoes crosstalk with inflammatory cytokines to exacerbate brain damage following intracerebral haemorrhage in mice. J Neuroinflammation.

[CR25] Wang R, Li Q, He Y, Yang Y, Ma Q, Li C (2020). miR-29c-3p inhibits microglial NLRP3 inflammasome activation by targeting NFAT5 in Parkinson’s disease. Genes Cells.

[CR26] Xiong XY, Liu L, Yang QW (2016). Functions and mechanisms of microglia/macrophages in neuroinflammation and neurogenesis after stroke. Prog Neurobiol.

[CR27] Yao L, Ye Y, Mao H, Lu F, He X, Lu G, Zhang S (2018). MicroRNA-124 regulates the expression of MEKK3 in the inflammatory pathogenesis of Parkinson’s disease. J Neuroinflammation.

[CR28] Yin H, Song S, Pan X (2017). Knockdown of miR-155 protects microglia against LPS-induced inflammatory injury via targeting RACK1: a novel research for intracranial infection. J Inflamm (Lond).

[CR29] Zheng X, Huang H, Liu J, Li M, Liu M, Luo T (2018). Propofol attenuates inflammatory response in LPS-activated microglia by regulating the miR-155/SOCS1 pathway. Inflammation.

[CR30] Zheng Y, Shang F, An L, Zhao H, Liu X (2018). NOD2-RIP2 contributes to the inflammatory responses of mice in vivo to Streptococcus pneumoniae. Neurosci Lett.

[CR202] Zhou Y, Hu L, Tang W, Li D, Ma L, Liu H, Zhang S, Zhang X, Dong L, Shen X (2021). Hepatic NOD2promotes hepatocarcinogenesis via a RIP2-mediated proinflammatory response and a novel nuclear autophagymediatedDNA damage mechanism. J Hematol Oncol.

