# Supplementary material for: MiR-200c-3p inhibits LPS-induced M1 polarization of BV2 cells by targeting RIP2
Source: Genes Genomics. 2022 Jan 10;44(4):477–86. doi: 10.1007/s13258-021-01210-z (PMC8921044; doi:10.1007/s13258-021-01210-z)
Supplement: Supplementary file 1 — Supplementary Material 1 (DOCX 16 KB) [file 13258_2021_1210_MOESM1_ESM.docx]

Dear Dr Yoon Shin Cho

On behalf of my co-authors, we thank you so much for giving us this opportunity to revise our manuscript. We deeply appreciate the editor and reviewers for their positive and helpful comments on our manuscript entitled “MiR-200c-3p inhibits LPS-induced M1 polarization of BV2 cells by targeting RIP2”. (Manuscript Number: GENG-D-21-01043).

We have thoughtfully taken these comments into account and have made revision which marked in red in this manuscript. We have already spared no effort to revise our manuscript according to these comments. Point to point to the reviewers as list in the response letter.

We are very grateful to you and reviewers for making comments on our paper. Looking forward to hearing from you.

Thank you and best regards.

Yours sincerely,

Yuhua Hu,

Email address: [huyuhua2000@163.com](mailto:huyuhua2000@163.com)
